# MiR-223 promotes the cisplatin resistance of human gastric cancer cells via regulating cell cycle by targeting FBXW7

**DOI:** 10.1186/s13046-015-0145-6

**Published:** 2015-03-26

**Authors:** Xiaoying Zhou, Wujuan Jin, Hongyan Jia, Jin Yan, Guoxin Zhang

**Affiliations:** Department of Gastroenterology, First Affiliated Hospital of Nanjing Medical University, Nanjing, 210029 China; First Clinical Medical College of Nanjing Medical University, Nanjing, 210029 China; Department of Digestive Endoscopy Center, First Affiliated Hospital of Nanjing Medical University, Nanjing, 210029 China

**Keywords:** miR-223, FBXW7, Cisplatin resistance, Gastric cancer

## Abstract

**Background:**

Increasing evidence showed that miRNAs serve as modulators of human cancer, either as oncogene or tumor suppressors. Cisplatin resistance is the most common cause of chemotherapy failure in gastric cancer (GC). However, the roles of miRNAs in cisplatin resistance of GC remain largely unknown. The aim of the study was to identify a novel miRNA/gene pathway that regulates the sensitivity of GC cells to cisplatin.

**Methods:**

In this study, we chose miR-223 by qRT-PCR analysis, the most significantly up-regulated miRNA in GC, to investigate its formation of DDP-resistant phenotype of GC cells and possible molecular mechanisms.

**Results:**

We found that miR-223 was most significantly up-regulated miRNA in DDP-resistant GC cells compared with parental GC cells. Besides, its expression was also significantly up-regulated in GC tissues. FBXW7 was identified as the direct and functional target gene of miR-223. Overexpression of FBXW7 could mimic the effect of miR-223 down-regulation and silencing of FBXW7 could partially reverse the effect of miR-223 down-regulation on DDP resistance of DDP-resistant GC cells. Besides, miR-223 and FBXW7 could affect the G1/S transition of cell cycle by altering some certain cell cycle regulators. Furthermore, miR-223 was found to be significantly up-regulated in H. pylori infected tissues and cells, suggesting that H. pylori infection may lead to GC development and DDP resistance.

**Conclusions:**

Our findings revealed the roles of miR-223/FBXW7 signaling in the DDP resistance of GC cells and targeting it will be a potential strategic approach for reversing the DDP resistance in human GC.

**Electronic supplementary material:**

The online version of this article (doi:10.1186/s13046-015-0145-6) contains supplementary material, which is available to authorized users.

## Introduction

Gastric cancer (GC) is the second leading cause of cancer-related deaths worldwide [1]. With an overall 5-year survival rate of only 20%, it becomes a major cause of both morbidity and mortality, where even resectable disease has a 50-90% risk of recurrence and death [2]. However, therapies often fail due to cancer cell multidrug resistance (MDR), which tends to develop after the initial rounds of treatment or before treatment begins (intrinsic MDR) [3]. The molecular mechanism underlying single or multidrug resistance to chemotherapeutic agents is complex and involves increases in drug efflux, insensitivity to drug-induced apoptosis and the enhancement of drug detoxification [4]. Although great efforts have been made to understand the mechanism underlying multidrug resistance, the current knowledge remains limited [5].

MicroRNAs (miRNAs) are a large class of endogenous non-coding RNAs, 21–23 nucleotides in length that regulate about 30% of human gene expression [6]. MiRNAs can function post-transcriptionally through imperfect base pairing with specific sequences in the 3’ untranslated regions (UTRs) of target mRNAs, leading to transcript degradation or translational inhibition [7]. Increasing evidence has shown that miRNAs have critical roles in the control of various human biological processes, such as development, angiogenesis, apoptosis and differentiation [8]. Increasing researches have shown the existence and importance of miRNAs in the evolution of anticancer drug resistance and miRNAs expression profiling can be correlated with the development of drug resistance, suggesting that the miRNAs-mediated form of drug resistance adds another molecular mechanism of drug resistance [9]. A couple of recent studies have reported the role of miRNAs in modulating GC or other tumor chemoresistance. Zhang et al. showed that miR-106a could promote chemoresistance of cisplatin resistant human GC cells by targeting RUNX3 [10]. Shang et al. showed that miR-508-5p could reverse chemoresistance of GC cells by targeting ABCB1 and ZNRD1 [11]. Zhou et al. identified that miR-33a is up-regulated in chemoresistant OS and that the miR-33a level is negatively correlated with the TWIST protein level [12]. These studies provided initial clues for miRNAs in regulating GC chemoresistance.

In the present study, we demonstrated that miR-223 could promote DDP resistance of GC cells via regulating G1/S cell cycle transition and apoptosis by targeting FBXW7. Thus, this report identifies novel signaling pathways and molecules as potential therapeutic targets for the treatment of DDP-resistant human GCs.

## Materials and methods

### Patients and samples

A total of 50 pairs of tumor and adjacent tissues were collected from GC patients who performed gastrectomy prior to any treatment at the First Affiliated Hospital of Nanjing Medical University during October 2013 and September 2014. The basic characteristics of the enrolled patients were listed in Additional file 1: Table S1. For the use of materials for research purposes, written informed consent was obtained from each patient. The consent procedure and study protocol were approved by the Medical Institutional Ethical Committee of first affiliated hospital of Nanjing Medical University.

### Cell culture and transfection

SGC-7901 and BGC-823 and their respective resistance cells were purchased from Shanghai Institute of Cell Biology (Shanghai, China). All cell lines were cultured in RPMI 1640 (GIBCO, Rockville, MD, USA) medium supplemented with 10% fetal bovine serum (FBS), 100 U/ml penicillin and 100 mg/ml streptomycin in humidified air at 37°C with 5% CO_2_. MiR-223 mimic or inhibitor or siRNA-FBXW7 and their negative controls were obtained from GenePharma (Shanghai, China). The open reading frame of FBXW7 that was generated by PCR was then inserted into the pcDNA 3.1 expression vector, which was named pcDNA-FBXW7. The recombinant vector was confirmed by the digestion analysis of restriction endonuclease and DNA sequencing. For ectopic expression of miR-223, miR-223 mimic or miR-NC vectors were purchased from GenePharm. The transfection was performed using Lipofectamine 2000 (Invitrogen, Carlsbad, CA, USA) according to the instructions.

### Quantitative real-time PCR

Total RNA was isolated using TRIzol reagent. The specific RT primers used were: miR-223:5’-GTCGTATCCAGTGCGTGTCGTGGAGTCGGCAATTGCACTGGATACGACAACTCA-3’ and U6:5’-CGCTTCACGAATTTGCGTGTCA-3’. RT was performed using PrimeScript™ RT reagent Kit (Takara, Otsu, Japan) according to the manufacturer’s instructions. PCR primers used were: miR-223 sense, 5’-CCGCTCGAGGAGCTTCCAGCTGAGCACTGGG-3’ and antisense, 5’-CGACGCGTTATTGCGCCCCCATCAGCACT-3’; U6 sense, 5’-CTCGCTTCGGCAGCACA-3’ and antisense, 5’-AACGCTTCACGAATTTGCGT-3’; FBXW7 Forward, 5’-GTCCCGAGAAGCGGTTTGATA-3’; Reverse, 5’-TGCTCAGGCACGTCAGAAAAG-3’; GAPDH sense, 5’-GCACCGTCAAGGCTGAGAAC-3’ and antisense, 5’-TGGTGAAGACGCCAGTGGA-3’. QRT-PCR was performed using SYBR Premix ExTaq (TaKaRa, Dalian, China) according to the manufacturer’s protocol.

### Western blot assay

The cells were lysed using the mammalian protein extraction reagent RIPA (Beyotime, Beijing, China). Approximately a 50 μg protein extraction was separated by 10% SDS-PAGE, transferred to 0.22 mm nitrocellulose (NC) membrane (Sigma), and incubated with specific antibodies. Autoradiograms were quantified by densitometry using Quantity One software (Bio-Rad, CA, USA). β-actin (diluted 1:1000) antibody was used as a control and rabbit anti-FBXW7 (1:1000 dilution), p14 (1:100 dilution), p16 (1:100 dilution), p21 (1:150 dilution), p27 (1:50 dilution), CDK2 (1:200 dilution), CDK4 (1:100 dilution), CDK6 (1:100 dilution), c-myc (1:150 dilution), CCND1 (1:100 dilution), CCND2 (1:150 dilution), CCND3 (1:100 dilution), CCNE1 (1:150 dilution), CCNE2 (1:50 dilution) were provided by Cell Signaling Technology (MA, USA).

### In vitro chemosensitivity assay

The in vitro chemosensitivity assay was determined by MTT assay. Briefly, cells were seeded into 96-well plates (3.5 × 10^3^ cells/well) and allowed to attach overnight. Cells were then treated with various concentrations of DDP. At 24 h, cell vitality was assessed using 0.5 mg/mL MTT (Sigma, MO, USA) solution. Approximately 4 h later, the medium was replaced with 150 μl dimethyl sulfoxide (DMSO, Sigma, MO, USA) and vortexed for 10 min. The absorbance at 490 nm (A490) of each well was read using a spectrophotometer. Each experiment was performed in triplicate.

### Colony formation assay

Cells were trypsinized to single cell suspensions and were seeded 6-well plates at 500/well. After 14 days culture in RPMI 1640 medium with FBS, the colonies were stained with crystal violet solution and the number of colonies was counted. Each experiment was performed in triplicate.

### Luciferase reporter assay

A FBXW7-3’UTR luciferase reporter was created. Briefly, the 3’UTR sequence of FBXW7 predicted to interact with miR-223 was amplified and cloned into the EcoRI and XhoI sites of pGL3-luc vector (Promega, Madison, WI, USA). The site-directed mutagenesis of the miR-223 target-site was carried out using Invitrogen (Californlia, USA). The constructs were sequenced and named pGL3-luc-FBXW7/3’-UTR-wt or pGL3-luc-FBXW7/3’-UTR-mut. For reporter assays, SGC-7901 cells were cultured in 24-well plates and each transfected with 100 ng of pGL3-luc-FBXW7/3’UTR-wt or pGL3-luc-FBXW7/3’UTR-mut and miR-223 mimics or inhibitor using Lipofectamine 2000 (Invitrogen, USA). 48 hours after transfection, cells were harvested and assayed with Dual-Luciferase Reporter Assay kit (Promega, USA) according to the manufacturer’s instructions.

### Flow cytometric analysis of cell cycle and apoptosis

For apoptosis analysis, cells were treated with various concentrations of DDP for 24 h, harvested and fixed with 2.5% (v/v) glutaraldehyde for 30 min. The rate of apoptosis was determined using Annexin V-FITC and PI staining by flow cytometry. For cell cycle analysis, cells were treated with various concentrations of DDP for 24 h, washed with ice-cold PBS and fixed with 70% (v/v) ethanol overnight at −20°C. Fixed cells were rehydrated in PBS for 10 min and subjected to PI/RNase staining followed by flow cytometric analysis using a FACScan instrument (Becton Dickinson, Mountain view, CA, US) and CellQuest software (Becton Dickinson, San Jose, CA, US).

### Statistical analysis

All experimental data were expressed as the Mean ± S.D. The significance of differences of clinical data according to miR-223 expression was determined by Student’s t-test. All analyses were performed with SPSS 17.0 (SPSS Inc, Chicago, IL, USA) for Windows. The significance level was set at P < 0.05.

## Results

### MiR-223 is up-regulated in tumor tissues and DDP-resistant GC cells

**Figure 1:**
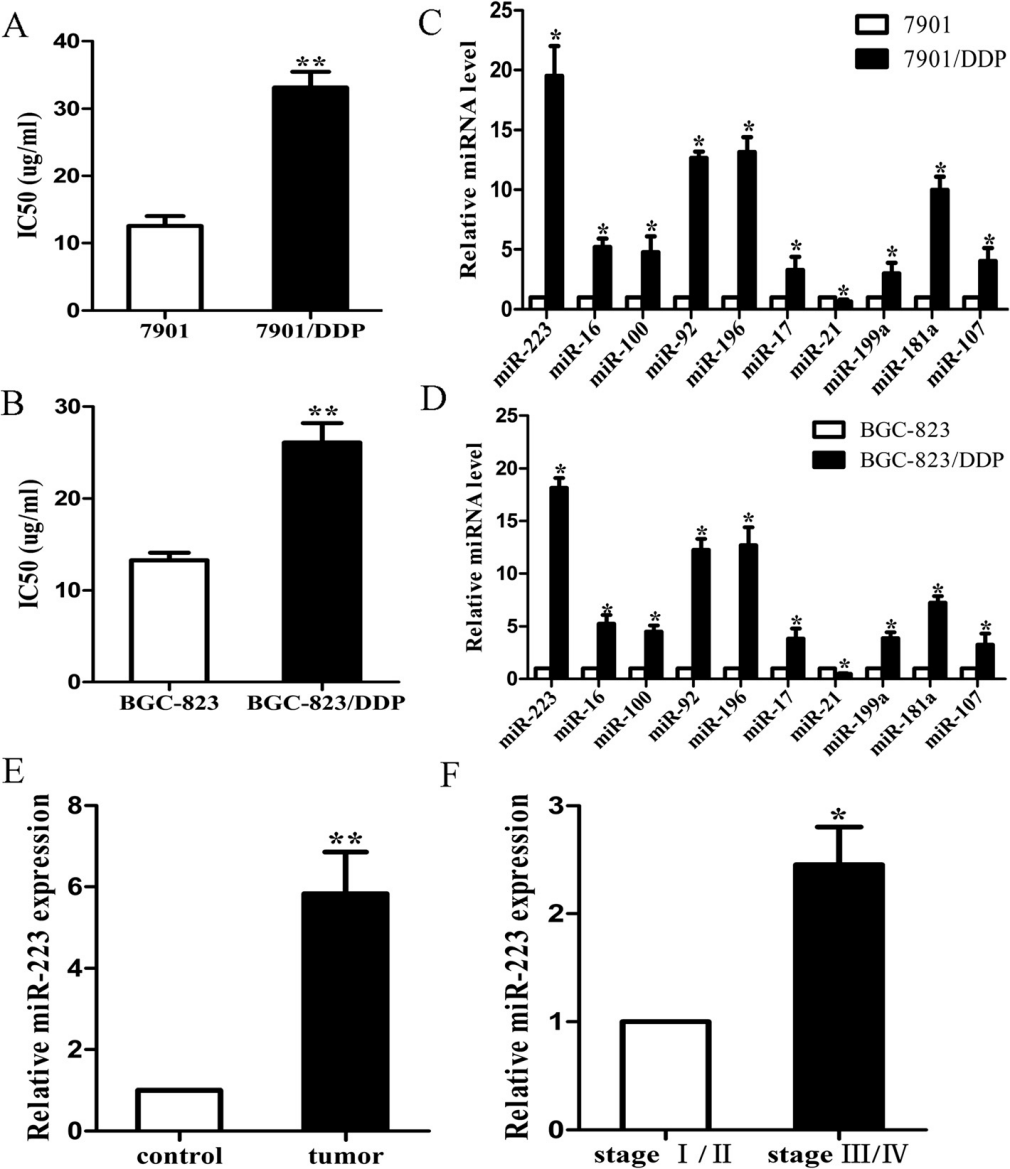
**miR-223 expression in cells and tissues. (A)** The IC_50_ level of 7901 and 7901/DDP; **(B)** The IC50 level of BGC-823 and BGC-823/DDP; **(C)** qRT-PCR detection of differential miRNAs expression in 7901 and 7901/DDP cells; **(D)** qRT-PCR detection of differential miRNAs expression in BGC-823 and BGC-823/DDP cells; **(E)** qRT-PCR detection of miR-223 in tumor and control tissues; **(F)** qRT-PCR detection of miR-223 in different stages of tumor tissues. (*p < 0.05, **p < 0.01).

To determine the correlation of dysregulated miRNAs with DDP resistance, frequently reported miRNAs related to GC were chosen to distinguish the difference between resistance and sensitivity of GC cells [13-22]. We detected the IC_50_ of sensitive and resistance cells first and found that the IC_50_ of 7901/DDP (Figure 1A) or BGC-823/DDP (Figure 1B) was significantly higher than that in respective sensitive cells. Then, we found that a panel of miRNAs was dysregulated in 7901/DDP cells compared with 7901 cells. Among the 10 miRNAs, miR-223 was found to be most significantly changed in 7901/DDP cells (P < 0.01) (Figure 1C). The result was similar in another GC cell line (P < 0.01; Figure 1D). We co-cultured 7901 and BGC-823 cells with different MOIs of H. pylori and found that miR-223 expression increased with MOIs of H. pylori infection (Additional file 2: Figure S1A). We also detected miR-223 expression in tumor and control tissues and found that miR-223 expression was significantly higher in tumors (Figure 1E, p < 0.05) and it expressed especially higher in stage III/IV patients (Figure 1F, p < 0.05). We further divided the patients into H. pylori infected and non-infected groups and found that miR-223 was significantly higher in H. pylori infected tumor and control adjacent tissues (Additional file 2: Figure S1B). Taken together, these data suggested that up-regulation of miR-223 might have important roles in the development of GC and DDP resistance in GC cells, especially under H. pylori infection state.

### Effect of miR-223 expression on sensitivity of GC cells to cisplatin in vitro

**Figure 2:**
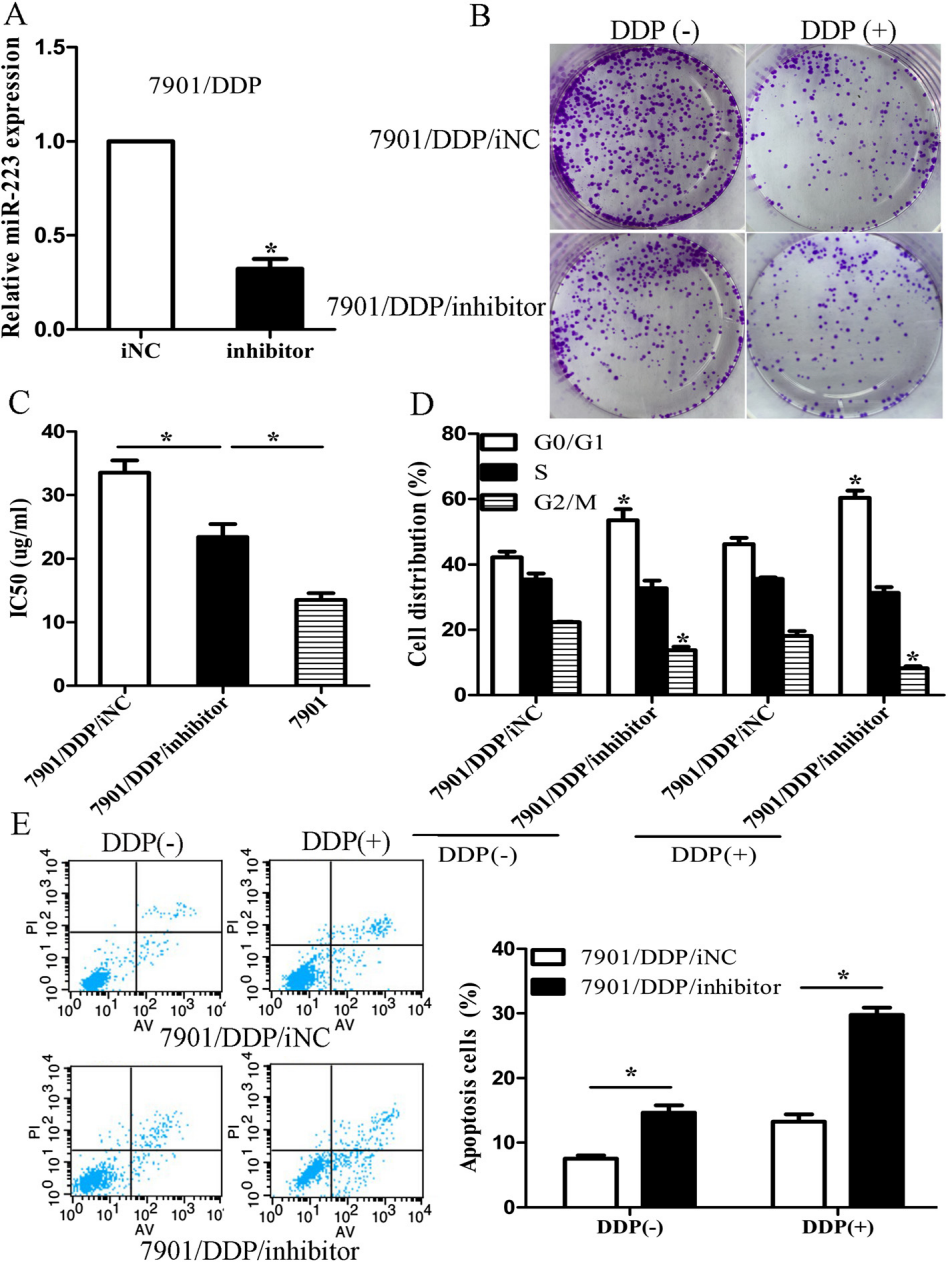
**Down-regulation of miR-223 significantly increases the sensitivity of 7901/DDP cells to DDP. (A)** Forty-eight hours after transfection with miR-223 inhibitor or inhibitor NC, qRT-PCR detection of miR-223 expression in cells. U6 was used as an internal control; **(B)** 7901/DDP cells transfected with miR-223 inhibitor show more DDP sensitivity than cells transfected with inhibitor NC. Indicated miR-223 inhibitor or inhibitor NC-transfected 7901/DDP cells were plated in triplicate and exposed to DDP (0.0 and 4.0ug/ml). The forci formation was indicated; **(C)** MTT analysis of the IC_50_ values of DDP in miR-224 inhibitor- or inhibitor NC-transfected 7901/DDP and parental 7901 cells; **(D)** Flow cytometric analysis of cell cycle in inhibitor NC- or miR-224 inhibitor-transfected 7901/DDP cells combined with DDP (0.0 and 4.0 ug/ml); **(E)** Flow cytometric analysis of apoptosis in miR-224 inhibitor- or inhibitor NC-transfected 7901/DDP combined with DDP (0.0 and 4.0 ug/ml); Data are expressed as the mean ± S.D. of three individual experiments. *p < 0.05.

To investigate the roles of miR-223 in the DDP resistance, miR-223 inhibitor or inhibitor NC (iNC) was transiently transfected into 7901/DDP and BGC-823/DDP cells. Forty-eight hours after transfection, qRT-PCR assay was performed and indicated that miR-223 expression was significantly decreased after inhibitor transfection (p < 0.05) (Figure 2A). Then, we determined the effect of miR-223 inhibitor on colony formation of 7901/DDP cells when exposed to DDP treatment (0 and 4 ug/ml). As shown in Figure 2B and Additional file 2: Figure S1C, the capacity of colony formation in miR-223 inhibitor transfected 7901/DDP and BGC-823/DDP cells was significantly decreased compared with iNC transfected cells (p < 0.05). The IC_50_ value of DDP in miR-223 inhibitor transfected 7901/DDP or BGC-823/DDP cells was also significantly reduced substantially (p < 0.05). Meantime, the IC_50_ value in miR-223 inhibitor transfected 7901/DDP or BGC-823/DDP cells still showed higher levels than that in respective sensitive cells, suggesting that inhibiting miR-223 expression could only partially reverse the DDP-resistant GC cells to DDP-sensitive phenotype (Figure 2C). Next, we analyzed the effects of miR-223 inhibitor on cell cycle and apoptosis of 7901/DDP cells when exposed to DDP treatment by flow cytometry. Compared with iNC transfected cells, the percent of 7901/DDP or BGC-823/DDP cells in G0/G1 phase after miR-223 inhibitor transfected increased, while cells in S phase was decreased with different doses of DDP (Figure 2D). Also, miR-223 could significantly increase DDP-induced apoptosis of resistant cells (Figure 2E). Therefore, down-regulation of miR-223 could reverse the DDP resistance of resistant GC cells by inducing cell arrest in G0/G1 phase and enhanced apoptosis when exposed to DDP treatment.

**Figure 3:**
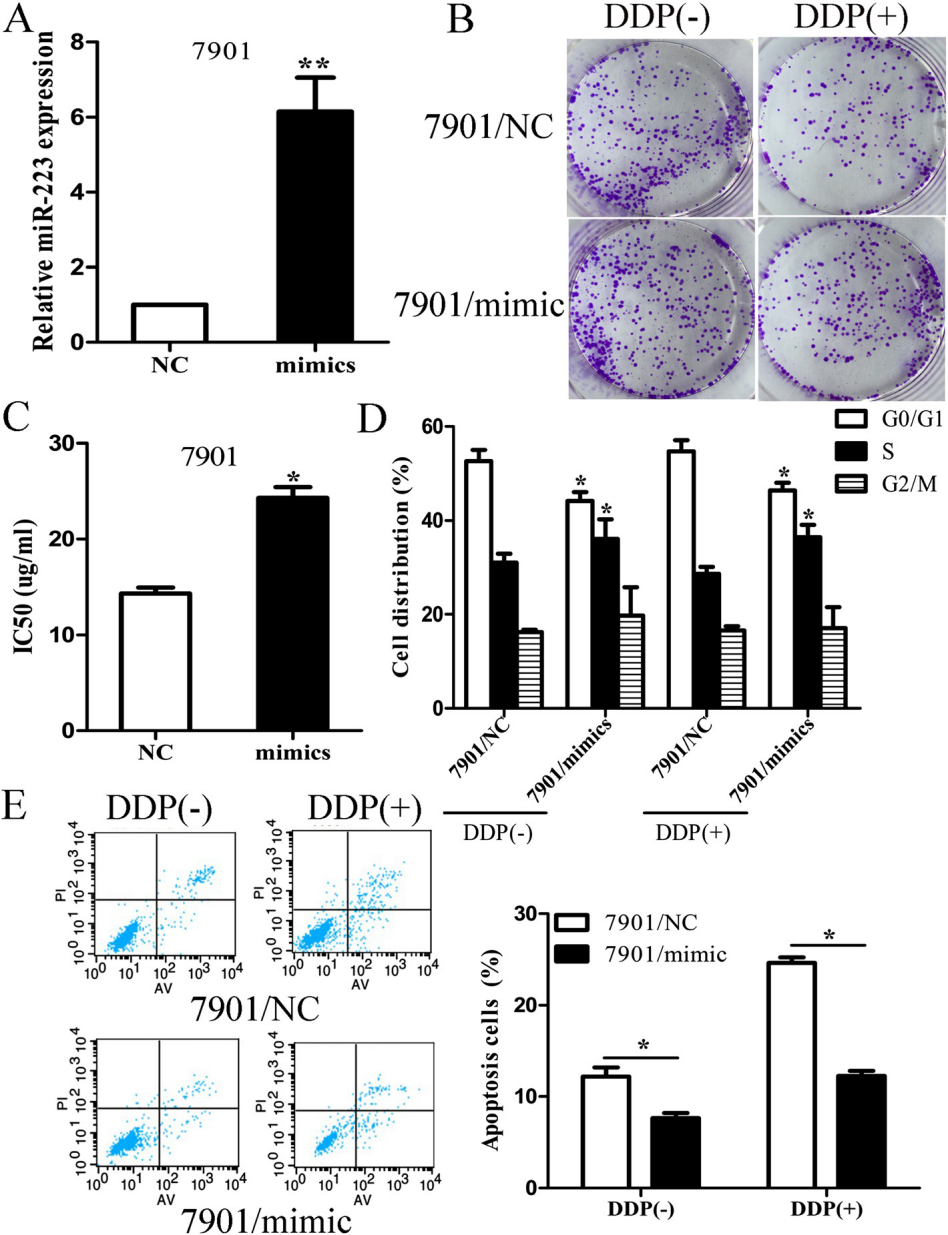
**Up-regulation of miR-223 significantly reduces the sensitivity of 7901 cells to DDP. (A)** qRT-PCR detection of miR-223 expression in miR-223 mimic or NC-tranfected 7901 cells. U6 was used as an internal control; **(B)** 7901/miR-223 cells show less DDP sensitivity than 7901/NC cells. Indicated 7901/miR-223 or 7901/NC cells were plated in triplicate and exposed to DDP (0 and 2.0 ug/ml). The forci formation was indicated; **(C)** MTT analysis of the IC_50_ values of DDP in 7901/miR-224 or 7901/NC cells; **(D)** Flow cytometric analysis of cell cycle in 7901/miR-223 or 7901/NC cells combined with DDP (0 and 2.0 ug/ml); **(E)** Flow cytometric analysis of apoptosis in 7901/miR-223 or 7901/NC cells combined with DDP (0 and 2.0 ug/ml); Data are expressed as the mean ± S.D. of three individual experiments. (*p < 0.05, **p < 0.01).

We further investigated the role of miR-223 over-expression on sensitive cells. We transient transfected miR-223 mimic or NC into 7901 or BGC-823 cells. qRT-PCR assay confirmed the up-regulated expression level of miR-223 in 7901/miR-223 and BGC-823/miR-223 cells, compared with that in 7901/NC or BGC-823/NC cells (Figure 3A). When exposed to DDP treatment (0 or 2 ug/ml), the capacity of colony formation in 7901/miR-223 or BGC-823/miR-223 cell was increased (p < 0.05) (Figure 3B and Additional file 2: Figure S1D). Compared with that in control cells, the IC_50_ value of DDP in 7901/miR-223 or BGC-823/miR-223 cells was increased (Figure 3C). Next, we analyzed the effects of miR-223 up-regulation on cell cycle and apoptosis of parental GC cells when exposed to DDP treatment by flow cytometry. Compared with control cells, the percent of 7901/miR-223 or BGC-823/miR-223 cells in G0/G1 phase decreased and the percentage of cells in S phase increased with different doses of DDP (Figure 3D). The apoptosis of 7901/miR-223 or BGC-823/miR-223 cells was significantly decreased (Figure 3E). Thus, up-regulation of miR-223 could increase the in vitro resistance of 7901 or BGC-823 cells to DDP.

### FBXW7 was a functional target of miR-223

**Figure 4:**
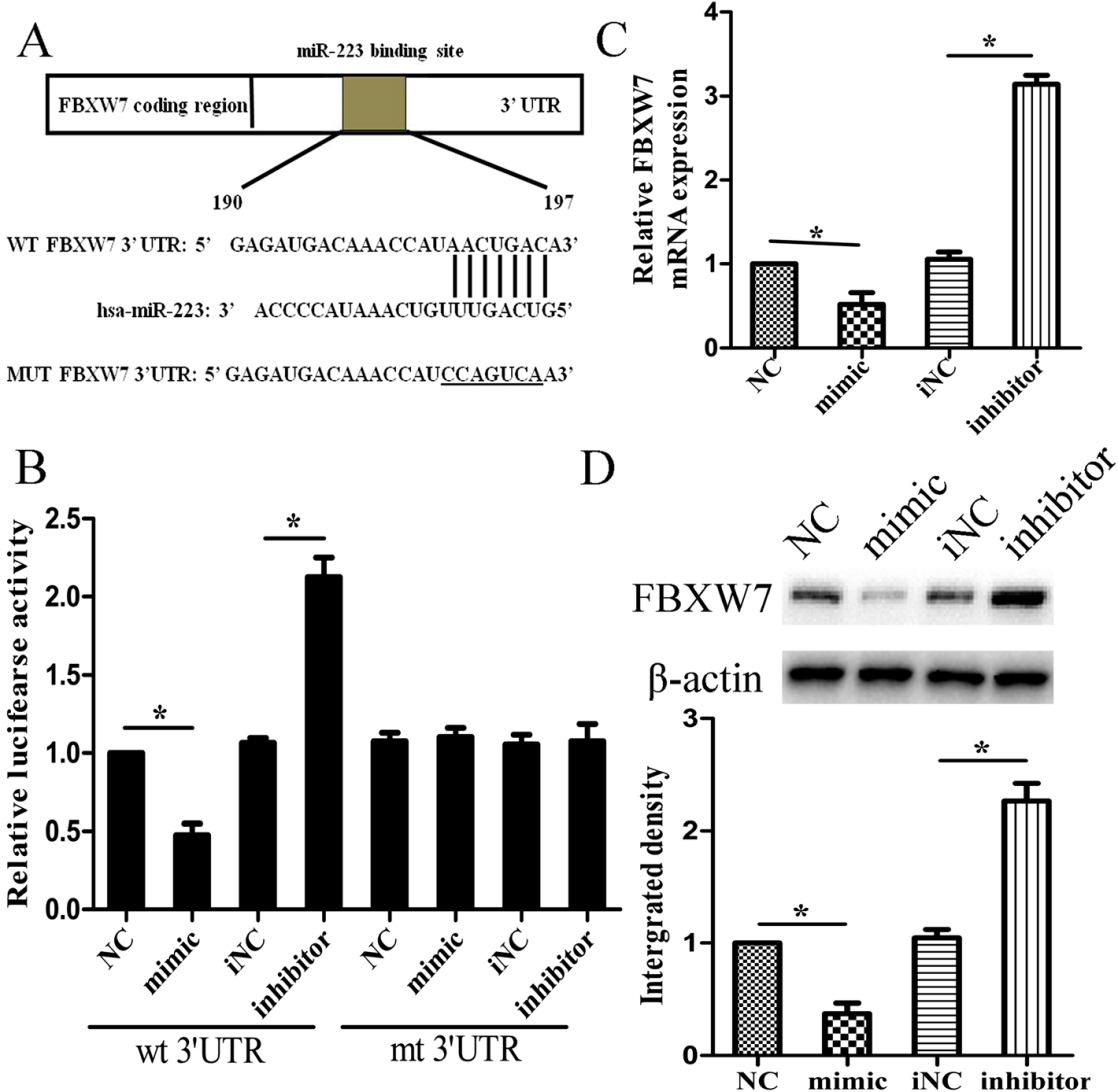
**MiR-223 binds to the 3’UTR of FBXW7 mRNA. (A)** Sequence of the miR-223-binding site within the FBXW7 3’UTR (190–197 bp) predicted with TargetScan and miRBase. Mutation was generated on the FBXW7 3’UTR sequence in the complementary site for the seed region of miR-223. A FBXW7 3’UTR fragment containing wild-type or mutant miR-223-binding sequence was cloned downstream of the luciferase reporter gene in pGL3-luc vector; **(B)** Relative luciferase activity was analyzed after wild-type or mutant 3’UTR reporter plasmids were co-transfected with miR-224 mimic or inhibitor in 7901 cells. The histogram shows the mean ± S.D. of the normalized luciferase activity from three independent experiments; **(C)** qRT-PCR detection of FBXW7 mRNA expression in 7901 cells transfected with miR-223 mimic or inhibitor. GAPDH was used as an internal control; **(D)** Western blot detection of FBXW7 protein expression in cells transfected with miR-223 mimic or inhibitor. β-actin was used as an internal control. Data are expressed as the mean ± S.D. of three individual experiments. *p < 0.05.

It is known that miRNAs exert their function by dysregulating their target genes expression. Thus, miR-223 can perform its resistance promoting function by inhibiting tumor suppressor targets. The targets of miR-223 were predicted through three publicly available algorithms (TargetScan, PicTar and miRanda), and FBXW7 was selected as a putative target in all three software. To determine whether the 3’UTR region of FBXW7 mRNA is a direct functional target of miR-223, we cloned FBXW7 3’UTR containing the potential binding site into the pGL3 vector to generate the pGL3-FBXW7-wt vector (Figure 4A). The luciferase activity was determined after co-transfecting pGL3-FBXW7-wt vector and miR-223 mimic or NC and the result showed that the luciferase activity was decreased by miR-223 mimics transfection (P < 0.05) when the wild type 3’UTR of FBXW7 was present, and the activity was increased significantly (P < 0.05) when miR-223 was inhibited. No difference was found when transfecting with pGL3-FBXW7-mut vector (Figure 4B). Then, qRT-PCR and western blot assays were performed to analyze the effects of miR-223 expression change on FBXW7 mRNA and protein expression. The results showed that FBXW7 expressed significantly lower when transfecting with miR-223 mimic and higher when transfecting with miR-223 inhibitor, compared with respective controls (Figure 4C). Western blot assay showed similar results (Figure 4D). Therefore, miR-223 could negatively regulate the expression of FBXW7 by directly targeting FBXW7 3’UTR transcript.

**Figure 5:**
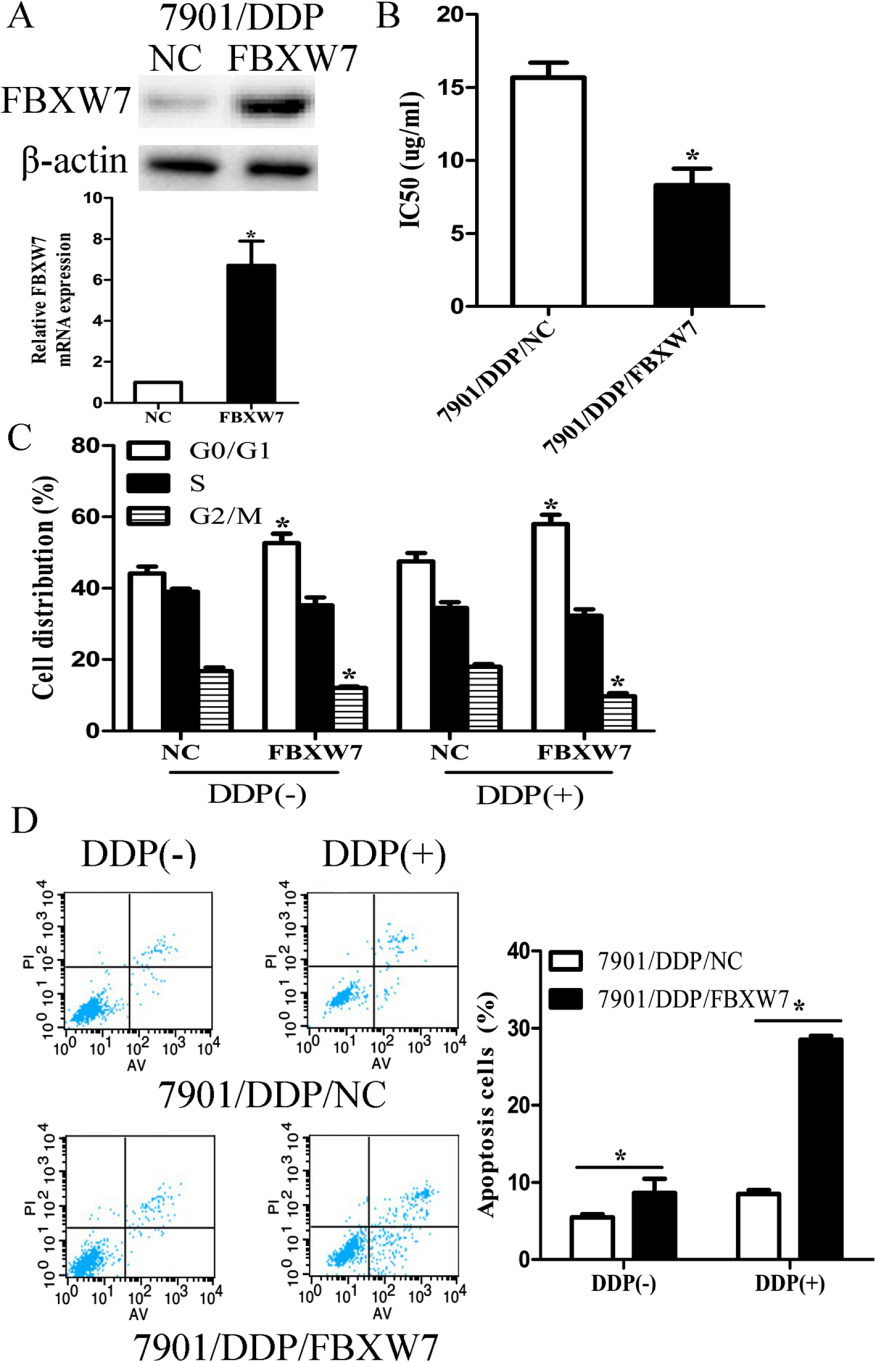
**Over-expression of FBXW7 could mimic the effect miR-223 inhibitor on the sensitivity of 7901/DDP cells. (A)** qRT-PCR and western blot detection of FBXW7 mRNA and protein expression in 7901/DDP/NC or 7901/DDP/FBXW7 cells; **(B)** MTT analysis of the IC_50_ values of DDP in 7901/DDP/NC or 7901/DDP/FBXW7 cells; **(C)** Flow cytometric analysis of cell cycle in 7901/DDP/NC or 7901/DDP/FBXW7 cells combined with DDP (0 and 2.0 ug/ml); **(D)** Flow cytometric analysis of apoptosis in 7901/DDP/NC or 7901/DDP/FBXW7 cells combined with DDP (0 and 2.0 ug/ml). Results represent the average of three independent experiments (mean ± S.D.). *p < 0.05.

To further investigate the roles of FBXW7 in DDP resistance, pcDNA/FBXW7 or pcDNA/NC was transiently transfected into 7901/DDP cells. Both qRT-PCR and western blot assays confirmed that the expression of FBXW7 mRNA and protein was significantly increased in 7901/DDP/FBXW7 cells (Figure 5A). Compared with 7901/DDP/NC cells, the IC_50_ value of DDP to 7901/DDP/FBXW7 cells was decreased significantly (Figure 5B). Flow cytometric analysis of cell cycle indicated that the percent of 7901/DDP/FBXW7 cells in G0/G1 phase of cell cycle increased and the percentage of cells in S phase decreased with different doses of DDP (Figure 5C). The increased apoptosis was observed in 7901/DDP/FBXW7 cells when exposed to different doses of DDP treatment (Figure 5D). These data indicated that FBXW7 up-regulation could increase the sensitivity of 7901/DDP cells to DDP. Further, we determined whether the up-regulation of FBXW7 could rescue the effects of miR-223 over-expression on the chemosensitivity of GC cells. qRT-PCR and western blot assays confirmed that pcDNA/FBXW7 could rescue the expression of FBXW7 mRNA and protein (Additional file 3: Figure S2A). Also, up-regulation of FBXW7 could rescue the increased IC_50_ value of DDP to 7901 cells induced by miR-223 over-expression (Additional file 3: Figure S2B). In addition, up-regulation of FBXW7 could reverse the effects of miR-223 overexpression on the G1/S transition (Additional file 3: Figure S2C) and DDP-induced apoptosis (Additional file 3: Figure S2D) of 7901 cells. Thus, up-regulation of FBXW7 could mimic the effect of miR-223 down-regulation on the chemo sensitivity of 7901/DDP cells and rescue the effect of miR-223 overexpression on the chemo sensitivity of 7901 cells.

**Figure 6:**
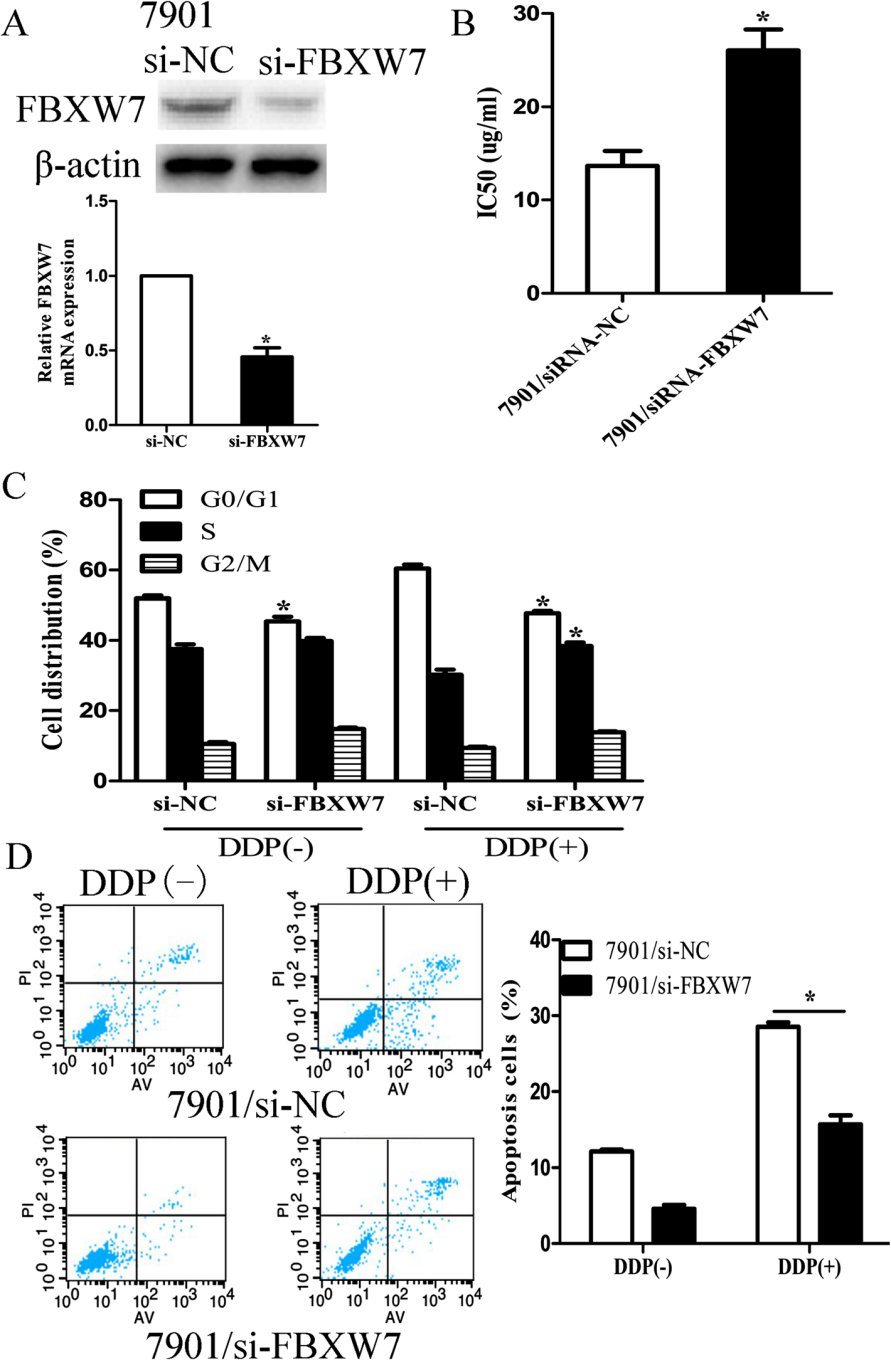
**siRNA-mediated down-regulation of FBXW7 could mimic effect of miR-223 up-regulation on sensitivity of 7901 cells. (A)** Forty-eight hours after transfection with siRNA/FBXW7 or siRNA/NC, qRT-PCR and western blot detection of FBXW7 mRNA and protein expression; **(B)** MTT analysis of the IC_50_ values of DDP in siRNA/NC or siRNA/FBXW7-transfected 7901 cells; **(C)** Flow cytometric analysis of cell cycle in siRNA/NC or siRNA/FBXW7-transfected 7901 cells combined with DDP (0 and 2 ug/ml); **(D)** Flow cytometric analysis of apoptosis in siRNA/NC or siRNA/FBXW7-transfected 7901 cells combined with DDP (0 and 2 ug/ml). Data are expressed as the mean ± S.D. of three individual experiments. *p < 0.05.

Finally, FBXW7 siRNA was transiently transfected into 7901 cells. Forty-eight hours after transfection, qRT-PCR and western blot assays showed that FBXW7 expression was significantly down-regulated in FBXW7 siRNA transfected cells (Figure 6A). Also, the IC_50_ value of DDP to 7901/siRNA/FBXW7 cells was increased compared with that to 7901/siRNA/control cells (Figure 6B). When 7901 cells were transfected with siRNA/FBXW7 combined with DDP treatment, flow cytometry was performed to detect cell cycle and apoptosis. Cell cycle analysis showed that siRNA mediated FBXW7 down-regulation could lead to the decreased G0/G1 phase rate and the increased S phase rate of 7901 cells when exposed to different doses of DDP treatment (Figure 6C). Compared with that in 7901/siRNA/control cells, the decreased apoptosis was observed in 7901/siRNA/FBXW7 cells when exposed to different doses of DDP treatment (Figure 6D). Thus, siRNA/FBXW7 could decrease the sensitivity of 7901 cells to DDP. We further determined whether FBXW7 down-regulation could rescue the effects of miR-223 down-regulation on the chemo sensitivity of 7901/DDP cells. Forty-eight hours after siRNA/FBXW7 and miR-223 inhibitor were co-transfected into 7901/DDP cells, qRT-PCR and western blot assays showed that siRNA/FBXW7 could recover the expression of FBXW7 mRNA and protein (Additional file 4: Figure S3A). Also, siRNA/FBXW7 could decrease the increased IC_50_ value of DDP to 7901/DDP cells induced by miR-223 inhibitor (Additional file 4: Figure S3B), and siRNA/FBXW7 could abrogate the effects of miR-223 inhibitor on the G1/S transition (Additional file 4: Figure S3C) and DDP-induced apoptosis (Additional file 4: Figure S3D) of 7901/DDP cells. Thus, down-regulation of FBXW7 could mimic the effect of miR-223 mimic on the chemo sensitivity of 7901 cells and rescue the effect of miR-223 inhibitor on the chemo sensitivity of 7901/DDP cells.

### MiR-223 and FBXW7 regulate the expression of G1/S transition in DDP-resistant GC cells

**Figure 7:**
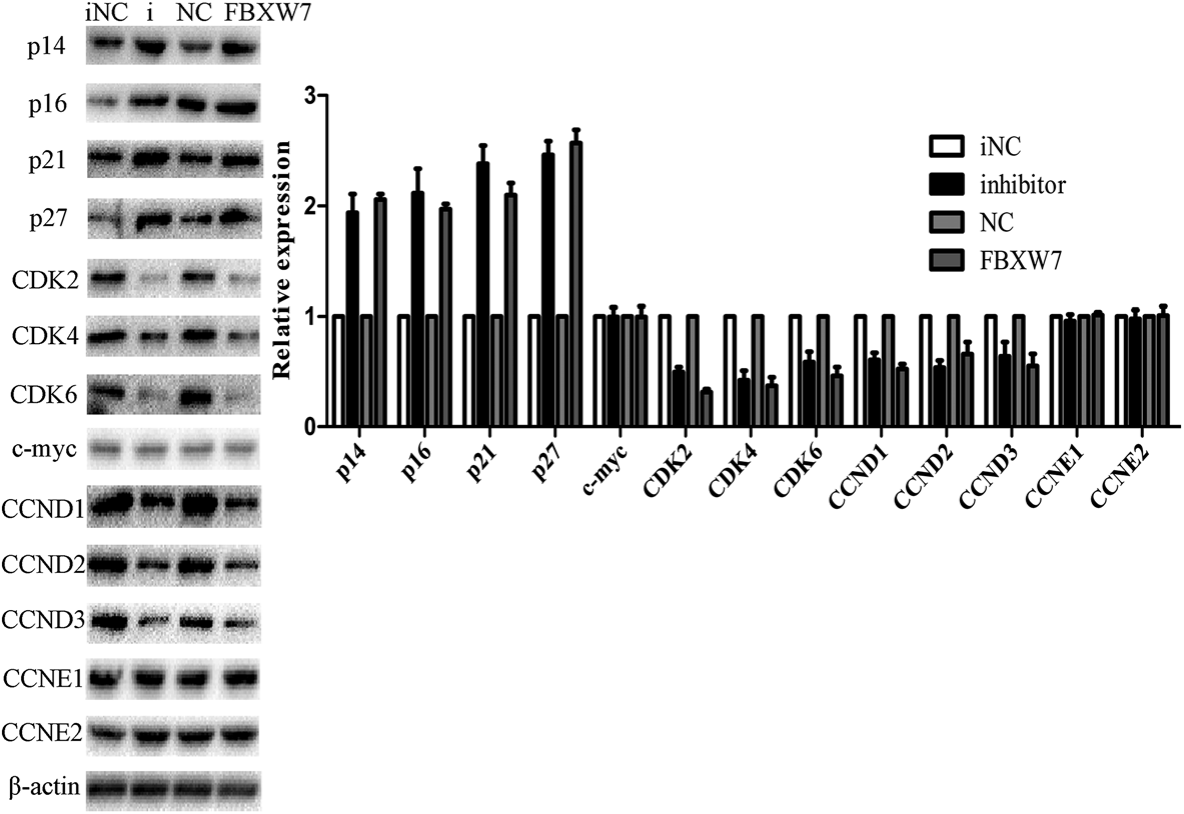
**Western blot detection of cell cycle-related protein expression.** Forty-eight hours after 7901/DDP cells were transfected with miR-223 inhibitor or pcDNA/FBXW7, cells were collected. Western blot detection of cell cycle-related proteins (p14, p16, p21, p27, CDK2, CDK4, CDK6, c-myc, CCND1, CCND2, CCND3, CCNE1 and CCNE2). β-actin protein was used as an internal control. Data are expressed as the mean ± S.D. of three individual experiments.

To further investigate the roles of FBXW7 in the miR-223-regulated cell cycle in DDP-resistant 7901 cells, we analyzed the expression of G1/S checkpoint related proteins in miR-223 inhibitor or pcDNA/FBXW7 transfected 7901/DDP cells, including CCND1, CCND2, CCND3, CCNE1, CCNE2, CDK2, CDK4, CDK6, p14, p16, p21, p27, c-myc (Figure 7). The expression of CDK2, CDK4, CDK6, CCND1, CCND2 and CCND3 was significantly down-regulated in miR-223 inhibitor or pcDNA/FBXW7-transfected 7901/DDP cells compared with control cells, while p14, p16, p21 and p27 were significantly up-regulated. However, the expression of c-myc, CCNE1 and CCNE2 showed no obvious difference between transfected cells and control cells. Our results indicated that miR-223 and FBXW7 might affect the G1/S transition of cell cycle.

### Expression of miR-223 was negatively correlated with FBXW7 expression in cells and tissues

**Figure 8:**
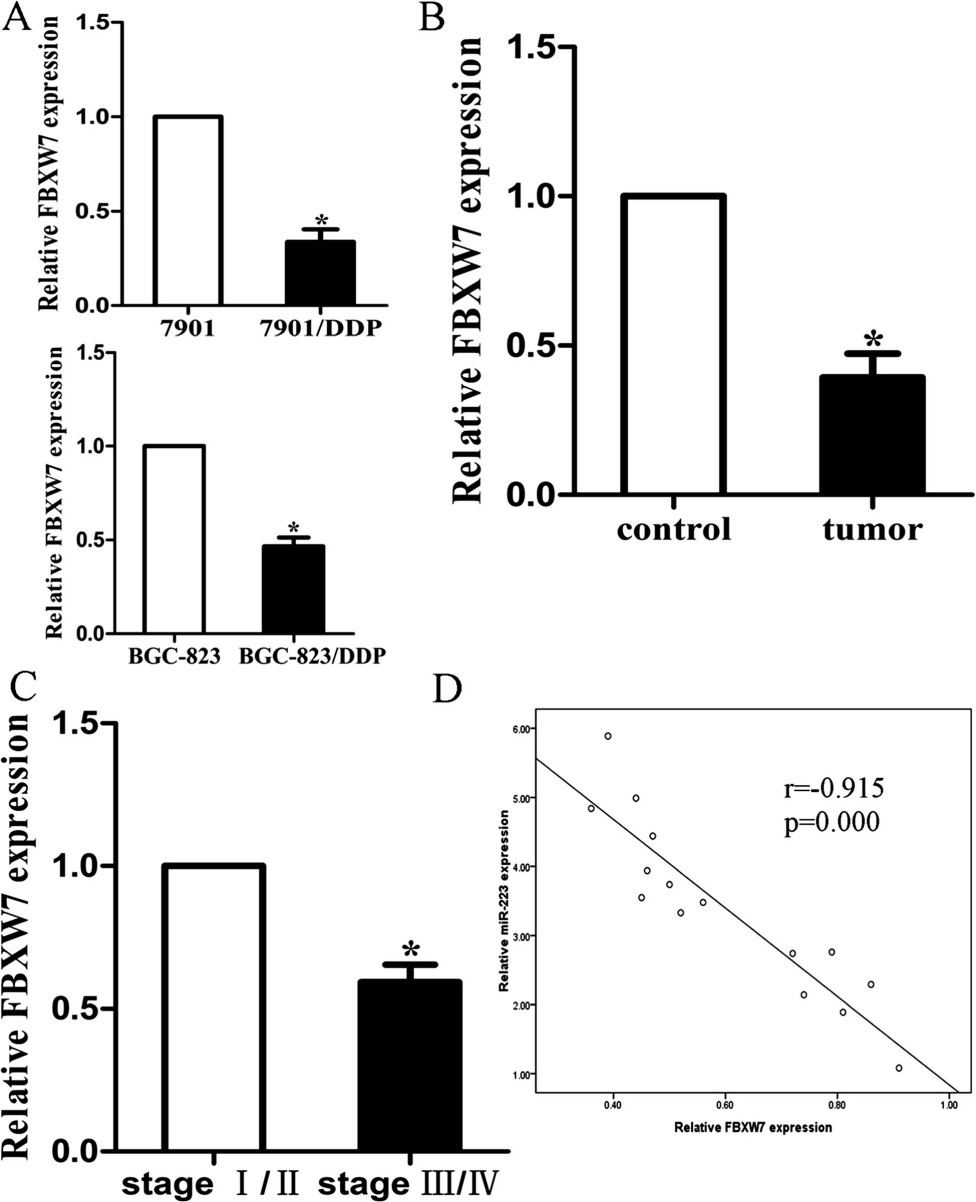
**Expression of miR-223 was negatively correlated with FBXW7 in cells and tissues. (A)** qRT-PCR detection of FBXW7 mRNA in 7901 and BGC-823 and their respective resistant cells; **(B)** qRT-PCR detection of FBXW7 mRNA in tumor and control tissues; **(C)** qRT-PCR detection of FBXW7 mRNA in different stages in tumor tissues; **(D)** A statistically significant inverse correlation between miR-223 and FBXW7 mRNA levels in 15 cases of GC tissues. (Spearman’s correlation analysis, r = −0.915, p = 0.000). Data are expressed as the mean ± S.D. of three individual experiments. Corresponding P values analyzed by Spearman correlation test are indicated.

We detected the expression level of FBXW7 mRNA in sensitive and resistant cells and found that FBXW7 expressed significantly lower in 7901/DDP or BGC-823/DDP cells (Figure 8A). The result was the same that FBXW7 expression was significantly lower in GC tissues compared with controls (Figure 8B). The relation between FBXW7 expression and clinicopathological features was listed in Additional file 5: Table S2. When we divided the patients into early and late stage, we found that the expression of FBXW7 was significantly lower in stage III/IV patients (Figure 8C). Then, we correlated FBXW7 with miR-223 expression in the same GC cells and specimens. A significant inverse correlation was observed (2-tailed Spearman's correlation, r = −0.915; P = 0.000) when FBXW7 mRNA levels were plotted against miR-223 expression (Figure 8D).

## Discussion

Cisplatin (DDP), a DNA damaging chemical, has been for many years used as a systematic chemotherapeutic agent for several human tumor types, including GC. It could increase cell death and apoptosis, arresting cells in G0/G1 phase [23]. Unfortunately, intrinsic or acquired tumor cell resistance to DDP severely limits its therapeutic efficacy. Multiple mechanisms have been proposed for the development of DDP resistance in GC, including the reduced intracellular accumulation of the drug, increased levels of glutathione and anti-apoptotic proteins, and decreased pro-apoptotic proteins [24]. Growing evidence has shown that miRNAs have regulatory roles in the pathogenesis of malignant tumors, through the suppression of genes involved in cell growth, differentiation, development, apoptosis, metastasis and chemo- or radio-resistance. It is likely, therefore, that they can also modulate sensitivity and resistance to anticancer drugs in substantial ways. However, the mechanisms responsible for chemotherapy resistance by miRNAs in GC have not been clearly identified.

MiR-223 has been known to be up-regulated in many human cancers, including colorectal cancer [25], lung cancer [26] and GC [27]. Specially, miR-223 was reported to be involved in trastuzumab induced resistance of GC [13]; however, it is still unclear whether miR-223 play a role in DDP induced resistance. The prognostic values of miR-223 in human cancers are also investigated [28]. These experimental data, taken together, support an important role of altered miR-223 during tumor progression and metastasis. Additionally, miR-223 was reported to be significantly up-regulated in H. pylori infected gastric tissues through miRNA array profiling. We found in the present study that H. pylori positive patients had a significantly higher proportion of miR-223 over-expression. In this study, we will investigate the emerging roles of miR-223 in DDP resistance of human GC cells. By loss-of-function studies, down-regulation of miR-223 could reverse the in vitro DDP resistance of DDP-resistant GC cells by affecting G1/S cell cycle transition and inducing apoptosis enhancement. Meanwhile, parental GC cell line transfecting with miR-223 mimic was established for gain-of-function studies. We showed that up-regulation of miR-223 could reduce the sensitivity of parental GC cells to DDP in vitro. Likewise, we also found that up-regulation of miR-223 could affect G1/S transition and reduce the DDP-induced apoptosis in parental GC cells. Importantly, to further explore the molecular mechanisms by which miR-223 exerts its function, the determination of its functional target gene is essential. Analyses using the TargetScan, PicTar and miRanda algorithms’ databases revealed that more than 100 genes were predicted to be the potential targets of miR-223. According to the functions of these genes and the effect of miR-223 on GC cells, FBXW7 was chosen as the interesting gene in further study. Our data clearly suggest that miR-223 promotes the DDP resistance of GC cells by directly targeting FBXW7. This conclusion is based on several pieces of evidence. First, miR-223 inhibitor significantly up-regulates the expression of FBXW7 mRNA and protein in 7901/DDP cells, whereas miR-223 mimic significantly down-regulates the expression of FBXW7 mRNA and protein in 7901 cells. Second, the luciferase activity assay indicated that miR-223 could bind the 3’-UTR of the FBXW7 transcript. Third, overexpression of FBXW7 could mimic the effect of miR-223 inhibitor in 7901/DDP cells, whereas silencing of FBXW7 could partially reverse the effect of miR-223 inhibitor in 7901 cells. Fourth, down-regulation of FBXW7 could mimic the effect of miR-223 mimic in 7901 cells, whereas up-regulation of FBXW7 could partially reverse the effect of miR-223 mimic in 7901/DDP cells. Finally, FBXW7 was negatively correlated with miR-223 in GC tissues. These data suggest that miR-223 targets FBXW7 and down-regulates its expression in GC.

FBXW7, also known as F-box and WD repeat domain-containing 7, has been found to be involved in numerous cellular processes including cell proliferation, apoptosis, cell cycle, differentiation and its expression change is related to tumor prognosis [29,30]. FBXW7 has been identified as a p53 target gene. In support of this notion, Fbxw7 was dramatically up-regulated by infection with adenovirus-mediated transfer of wild-type p53 into the p53-deficient cells [31]. It is considered as a p53-dependent tumor suppressor protein and leads to ubiquitination-mediated suppression of several oncoproteins including c-Myc, cyclin E, Notch, c-Jun and others [32,33]. In our study, however, we did not find c-myc protein change after FBXW7 transfection. C-myc mRNA was inhibited after FBXW7 overexpression and increased after FBXW7 knock down. This is probably due to the post-transcriptional regulation of the genes. MicroRNAs (miRNAs) including miR-27 [34], miR-25 [35] and miR-223 [36] have been reported to be involved in regulating the expression of FBXW7. Wang et al. reported that FBXW7 is a potential miR-27a target. Consistently, there is an inverse correlation between miR-27a expression and FBXW7 levels in human tumor samples. Lerner et al. further discovered that miR-27a suppresses FBXW7 during specific cell cycle phases [37]. Wertz et al. reported that FBXW7 inactivation and increased Mcl-1 levels promoted resistance to anti-tubulin chemotherapeutic agents and accelerated tumorigenesis. This group also reported that inhibition of Mcl-1 in FBXW7 null cells restored their sensitivity to taxol- and vincristine-induced cell death [38]. Collectively, these provided stronger evidence that the formation of DDP resistant phenotype of GC cells was mediated via FBXW7 inactivation.

In summary, our data establish a functional link that miR-223 and FBXW7 in GC, and show that miR-223 could promote DDP resistance in GC cells via regulating cell cycle and apoptosis by targeting FBXW7. Thus, in the future, miR-223/FBXW7 signature might predict the responses of GC patients to DDP-based chemotherapy and represent potential targets for therapeutic intervention.

## Additional files

Additional file 1: Table S1. Correlation between miR-223 expression and clinicopathological features of GC patients. The associations of miR-223 with clinicopathological characteristics of patients were detected by the two-tailed Student’s t-test; *P < 0.05 was considered statistically significant. Additional file 2: Figure S1. (A) miR-223 expression in different MOIs of H. pylori infected cells compared with controls; (B) miR-223 expression in H. pylori positive and negative tumor tissues; (C) Clone numbers in miR-223 inhibitor transfected 7901/DDP and BGC-823/DDP cells; (D) Clone numbers in miR-223 mimic transfected 7901/DDP and BGC-823/DDP cells. (*p < 0.05, **p < 0.01). Additional file 3: Figure S2. Over-expression of FBXW7 could rescue the effect of miR-223 up-regulation on the sensitivity of 7901 cells to DDP. (A) 48 h after 7901/miR-223 or 7901/NC cells co-transfected with pcDNA/FBXW7 vector, qRT-PCR and western blot detection of FBXW7 mRNA and protein expression; (B) MTT analysis of the IC_50_ values of DDP in 7901/miR-223 or 7901/NC cells or those cells co-transfected with pcDNA/FBXW7; (C) Flow cytometric analysis of cell cycle in 7901/miR-223 or 7901/NC cells or those cells co-transfected with pcDNA/FBXW7; (D) Flow cytometric analysis of apoptosis in 7901/miR-223 or 7901/NC cells or those cells co-transfected with pcDNA/FBXW7 combined with DDP treatment (4.0 μg/ml). Data are expressed as the mean ± S.D. of three individual experiments. Additional file 4: Figure S3. SiRNA-mediated down-regulation of FBXW7 could rescue the effect of miR-223 downregulation on the sensitivity of 7901/DDP cells to DDP. (A) 48 h after 7901/DDP cells were co-transfected with miR-223 inhibitor and siRNA/FBXW7, qRT-PCR and western blot detection of FBXW7 mRNA and protein expression; (B) MTT analysis of the IC_50_ values of DDP in miR-223 inhibitor and siRNA/FBXW7-transfected 7901/DDP cells; (C) Flow cytometric analysis of cell cycle in iNC or miR-223 inhibitor-transfected 7901/DDP cells or those cells co-transfected with siRNA/FBXW7. (D) Flow cytometric analysis of apoptosis in iNC or miR-223 inhibitor-transfected 7901/DDP cells or those cells co-transfected with siRNA/FBXW7 combined with DDP treatment (4.0 μg/ml). Data are expressed as the mean ± S.D. of three individual experiments. Additional file 5: Table S2. Correlation between FBXW7 expression and clinicopathological features of GC patients. The associations of FBXW7 with clinicopathological characteristics of patients were detected by the two-tailed Student’s t-test; *P < 0.05 was considered statistically significant.
