# Supplementary material for: MiR-223 promotes the cisplatin resistance of human gastric cancer cells via regulating cell cycle by targeting FBXW7
Source: J Exp Clin Cancer Res. 2015 Mar 26;34(1):28. doi: 10.1186/s13046-015-0145-6 (PMC4387683; doi:10.1186/s13046-015-0145-6)
Supplement: Additional file 1: Table S1. — Correlation between miR-223 expression and clinicopathological features of GC patients. The associations of miR-223 with clinicopathological characteristics of patients were detected by the two-tailed Student’s t-test; *P < 0.05 was considered statistically significant. [file 13046_2015_145_MOESM1_ESM.docx]

| Clinicopathological  factors | MiR-223 expression | | | *P*-value |
| --- | --- | --- | --- | --- |
|  | High (n=27) | | Low (n=23) |  |
| Sex |  |  | |  |
| Male | 18 | 15 | | 0.575 |
| Female | 9 | 8 | |  |
| Age (years) |  |  | |  |
| ≤60 | 10 | 7 | | 0.623 |
| >60 | 17 | 16 | |  |
| Smoking |  |  | |  |
| Non-smoker | 13 | 10 | | 0.741 |
| Smoker | 14 | 13 | |  |
| Drinking |  |  | |  |
| No | 19 | 16 | | 0.951 |
| Yes | 8 | 7 | |  |
| Clinical stage |  |  | |  |
| Ⅰ-Ⅱ | 5 | 11 | | 0.027* |
| III -IV | 22 | 12 | |  |
| H. pylori infection |  |  | |  |
| Positive | 19 | 8 | | 0.012* |
| Negative | 8 | 15 | |  |

**Table S1** Correlation between miR-223 expression and clinicopathological features of GC patients.

The associations of miR-223 with clinicopathological characteristics of patients were detected by the two-tailed Student’s *t*-test; **P*<0.05 was considered statistically significant.
