# Supplementary figures and images for: MiR-223 promotes the cisplatin resistance of human gastric cancer cells via regulating cell cycle by targeting FBXW7
Source: J Exp Clin Cancer Res. 2015 Mar 26;34(1):28. doi: 10.1186/s13046-015-0145-6 (PMC4387683; doi:10.1186/s13046-015-0145-6)

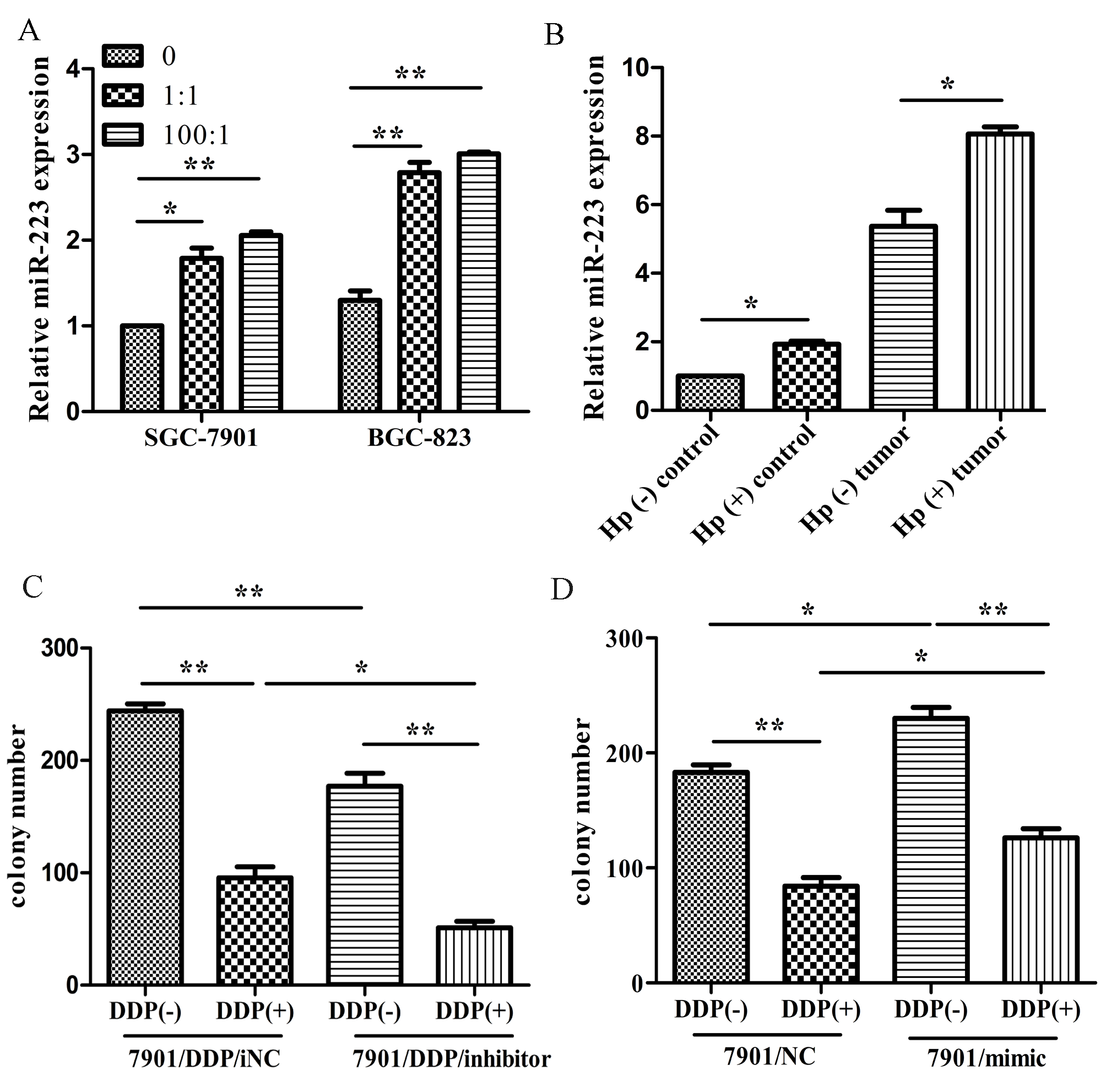

Supplement: Additional file 2: Figure S1. — (A) miR-223 expression in different MOIs of H. pylori infected cells compared with controls; (B) miR-223 expression in H. pylori positive and negative tumor tissues; (C) Clone numbers in miR-223 inhibitor transfected 7901/DDP and BGC-823/DDP cells; (D) Clone numbers in miR-223 mimic transfected 7901/DDP and BGC-823/DDP cells. (*p < 0.05, **p < 0.01). [file 13046_2015_145_MOESM2_ESM.tiff]

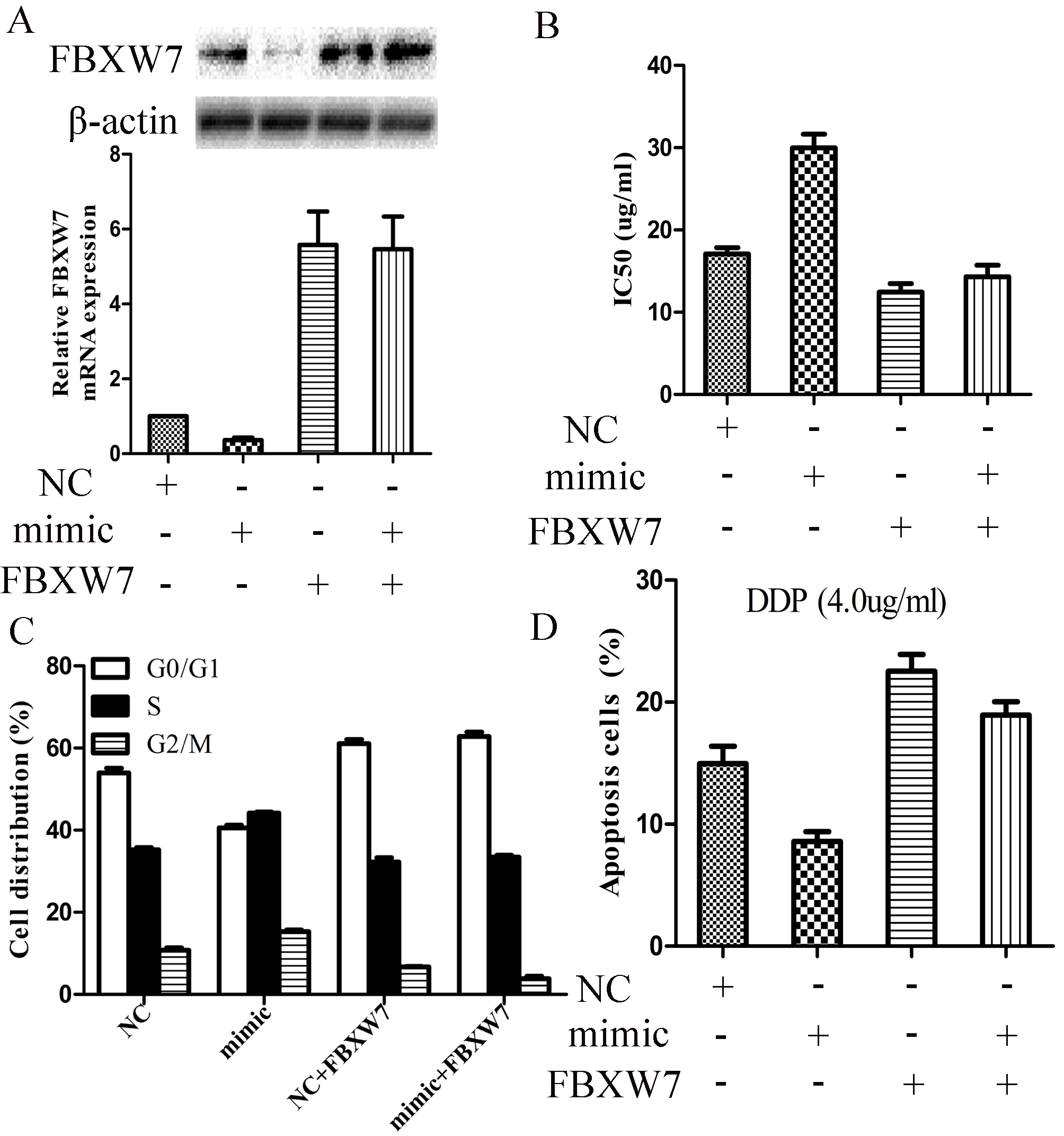

Supplement: Additional file 3: Figure S2. — Over-expression of FBXW7 could rescue the effect of miR-223 up-regulation on the sensitivity of 7901 cells to DDP. (A) 48 h after 7901/miR-223 or 7901/NC cells co-transfected with pcDNA/FBXW7 vector, qRT-PCR and western blot detection of FBXW7 mRNA and protein expression; (B) MTT analysis of the IC50 values of DDP in 7901/miR-223 or 7901/NC cells or those cells co-transfected with pcDNA/FBXW7; (C) Flow cytometric analysis of cell cycle in 7901/miR-223 or 7901/NC cells or those cells co-transfected with pcDNA/FBXW7; (D) Flow cytometric analysis of apoptosis in 7901/miR-223 or 7901/NC cells or those cells co-transfected with pcDNA/FBXW7 combined with DDP treatment (4.0 μg/ml). Data are expressed as the mean ± S.D. of three individual experiments. [file 13046_2015_145_MOESM3_ESM.tiff]

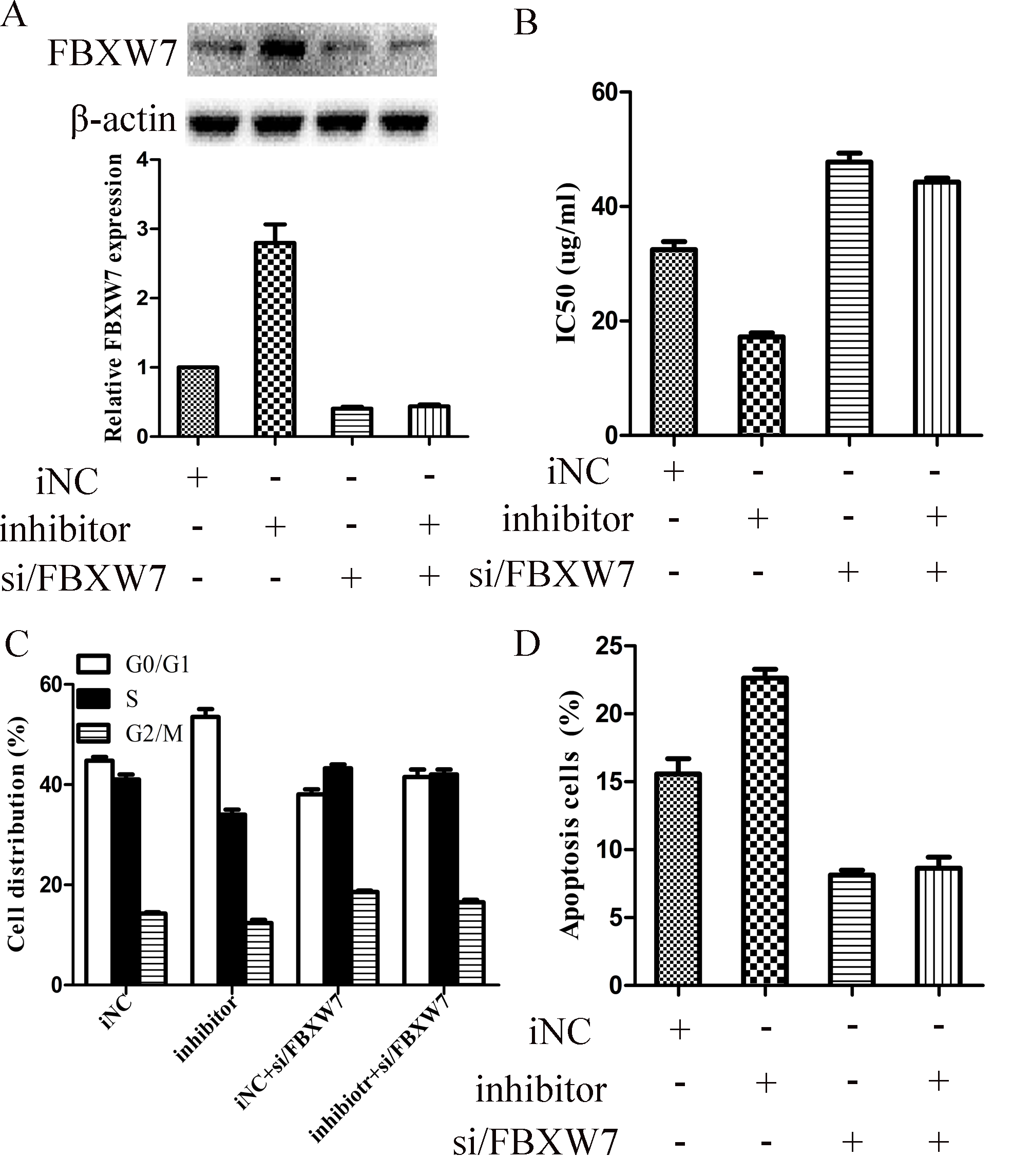

Supplement: Additional file 4: Figure S3. — SiRNA-mediated down-regulation of FBXW7 could rescue the effect of miR-223 downregulation on the sensitivity of 7901/DDP cells to DDP. (A) 48 h after 7901/DDP cells were co-transfected with miR-223 inhibitor and siRNA/FBXW7, qRT-PCR and western blot detection of FBXW7 mRNA and protein expression; (B) MTT analysis of the IC50 values of DDP in miR-223 inhibitor and siRNA/FBXW7-transfected 7901/DDP cells; (C) Flow cytometric analysis of cell cycle in iNC or miR-223 inhibitor-transfected 7901/DDP cells or those cells co-transfected with siRNA/FBXW7. (D) Flow cytometric analysis of apoptosis in iNC or miR-223 inhibitor-transfected 7901/DDP cells or those cells co-transfected with siRNA/FBXW7 combined with DDP treatment (4.0 μg/ml). Data are expressed as the mean ± S.D. of three individual experiments. [file 13046_2015_145_MOESM4_ESM.tiff]
