# Supplementary material for: MiR-223 promotes the cisplatin resistance of human gastric cancer cells via regulating cell cycle by targeting FBXW7
Source: J Exp Clin Cancer Res. 2015 Mar 26;34(1):28. doi: 10.1186/s13046-015-0145-6 (PMC4387683; doi:10.1186/s13046-015-0145-6)
Supplement: Additional file 5: Table S2. — Correlation between FBXW7 expression and clinicopathological features of GC patients. The associations of FBXW7 with clinicopathological characteristics of patients were detected by the two-tailed Student’s t-test; *P < 0.05 was considered statistically significant. [file 13046_2015_145_MOESM5_ESM.docx]

**Table S2** Correlation between FBXW7 expression and clinicopathological features of GC patients.

| Clinicopathological  factors | FBXW7 expression | | | *P*-value |
| --- | --- | --- | --- | --- |
|  | High (n=20) | | Low (n=30) |  |
| Sex |  |  | |  |
| Male | 12 | 21 | | 0.465 |
| Female | 8 | 9 | |  |
| Age (years) |  |  | |  |
| ≤60 | 7 | 10 | | 0.903 |
| >60 | 13 | 20 | |  |
| Smoking |  |  | |  |
| No | 8 | 15 | | 0.487 |
| Yes | 12 | 15 | |  |
| Drinking |  |  | |  |
| No | 15 | 20 | | 0.529 |
| Yes | 5 | 10 | |  |
| Clinical stage |  |  | |  |
| Ⅰ-Ⅱ | 5 | 11 | | 0.386 |
| III -IV | 15 | 19 | |  |
| Tumor depth |  |  | |  |
| Mucous/submucous | 14 | 11 | | 0.021**^*^** |
| Muscle/serosal | 6 | 19 | |  |
| Nodal metastasis |  |  | |  |
| No | 20 | 30 | | NA**^#^** |
| Yes | 0 | 0 | |  |
| Tumor histology |  |  | |  |
| Well-differentiated | 12 | 12 | | 0.166 |
| Poor- differentiated | 8 | 18 | |  |
| H. pylori infection |  |  | |  |
| Positive | 10 | 17 | | 0.643 |
| Negative | 10 | 13 | |  |
| miR-223 expression |  |  | |  |
| High | 6 | 21 | | 0.005**^*^** |
| Low | 14 | 9 | |  |

The associations of FBXW7 with clinicopathological characteristics of patients were detected by the two-tailed Student’s *t*-test; **P*<0.05 was considered statistically significant; ^#^NA: not applicable
